# Impact of organ motion on volumetric and dosimetric parameters in stomach lymphomas treated with intensity‐modulated radiotherapy

**DOI:** 10.1002/acm2.12681

**Published:** 2019-08-10

**Authors:** Yusuke Uchinami, Ryusuke Suzuki, Norio Katoh, Hiroshi Taguchi, Koichi Yasuda, Naoki Miyamoto, Yoichi M. Ito, Shinichi Shimizu, Hiroki Shirato

**Affiliations:** ^1^ Department of Radiation Oncology Hokkaido University Hospital Sapporo Japan; ^2^ Global Station for Quantum Medical Science and Engineering, Global Institution for Collaborative Research and Education (GI‐CoRE) Hokkaido University Sapporo Japan; ^3^ Department of Radiation Medicine, Faculty of Medicine Hokkaido University Sapporo Japan; ^4^ Department of Statistical Data Science The Institute of Statistical Mathematics Tokyo Japan; ^5^ Department of Radiation Medical Science and Engineering, Graduate School of Medicine Hokkaido University Sapporo Japan

**Keywords:** interplay effects, organ motion, stomach IMRT, stomach lymphoma

## Abstract

**Purpose:**

Interplay effects may influence dose distributions to a moving target when using dynamic delivery techniques such as intensity‐modulated radiotherapy (IMRT). The aim of this study was to evaluate the impact of organ motion on volumetric and dosimetric parameters in stomach lymphomas treated with IMRT.

**Methods:**

Ten patients who had been treated with IMRT for stomach lymphomas were enrolled. The clinical target volume (CTV) was contoured as the whole stomach. Considering interfractional uncertainty, the internal target volume (ITV) margin was uniformly 1.5 cm to the CTV and then modified based on the 4DCT images in case of the large respiratory motion. The planning target volume (PTV) was created by adding 5 mm to the ITV. The impact of organ motion on the volumetric and dosimetric parameters was evaluated retrospectively (4D simulation). The organ motion was reproduced by shifting the isocenter on the radiation treatment planning system. Several simulation plans were created to test the influence of the beam‐on timing in the respiration cycle on the dose distribution. The homogeneity index (HI), volume percentage of stomach covered by the prescribed dose (V_p_), and D_99_ of the CTV were evaluated.

**Results:**

The organ motion was the largest in the superior‐inferior direction (10.1 ± 4.5 mm [average ± SD]). Stomach volume in each respiratory phase compared to the mean volume varied approximately within a ± 5% range in most of the patients. The PTV margin was sufficiently large to cover the CTV during the IMRT. There was a significant reduction in V_p_ and D_99_ but not in HI in the 4D simulation in free‐breathing and multiple fractions compared to the clinically‐used plan (*P *< 0.05) suggesting that interplay effects deteriorate the dose distribution. The absolute difference of D_99_ was less than 1% of the prescribed dose.

**Conclusions:**

There were significant interplay effects affecting the dose distribution in stomach IMRT. The magnitude of the dose reduction was small when patients were treated on free‐breathing and multiple fractions.

**Abbreviations**
IMRT: intensity‐modulated radiotherapy
3D‐CRT: three‐dimensional conformal radiotherapy
3DCT: three‐dimensional computed tomography
4DCT: four‐dimensional CT
fb3DCT: free‐breathing CT images
3DCT_r%_: reconstructed 3DCT from 4DCT scan at a r% phase of a respiratory cycle
fus3DCT: fusion image set of the 3DCT_r%_ fused with fb3DCT
F3D plan: the clinically used IMRT plan
ISO_3D_: the crossing point of the central axis of the clinically used radiation beams
POI_3D_: the centroid of the contoured stomach in the fb3DCT
ISO_r%_: the assumed isocenter at the r% phase
POI_r%_: the centroid of the contoured stomach for each 3DCT_r%_
S4D simulation: simple 4D simulation
R4D simulation: random 4D simulation

## 1 INTRODUCTION

Radiation treatment is an important treatment option for gastric neoplastic diseases. Radiotherapy is accepted as a first‐line treatment for localized gastric mucosa‐associated lymphoid tissue (MALT) lymphomas which are refractory to Helicobacter pylori eradication. In MALT lymphomas, moderate dose radiotherapy is reported to be adequate to achieve excellent local control.^1^, ^2^, ^3^ In early‐stage primary gastric diffuse large B‐cell lymphomas (DLBCL), radiotherapy is also used to achieve locoregional control after chemotherapy.^4^, ^5^ In the field of gastric adenocarcinomas, radiotherapy or chemoradiotherapy has also been actively studied in preoperative and postoperative settings.^6^, ^7^ One of the challenges in precisely irradiating the stomach is its unique and irregular shape. At the same time, the accurate radiotherapy is essential to achieve satisfactory therapeutic effects and decrease adverse events.

Intensity‐modulated radiotherapy (IMRT) is widely used to achieve better dose conformity for the target volume and dose sparing of organs at risk (OAR) than is possible in three‐dimensional conformal radiotherapy (3D‐CRT). Good candidates for IMRT are for target volumes adjacent to critical organs. Because the stomach is surrounded by a number of critical organs such as kidneys, liver, colon, and the left lung, IMRT is considered to be advantageous to reduce the radiation dose to these OAR. Inaba et al. studied 17 patients with gastric lymphomas and demonstrated that IMRT displayed advantages in reducing the radiation dose to abdominal organs when compared with 3D‐CRT.^8^ Using four‐dimensional CT (4DCT) images for the stomach, Van Der Geld et al. also reported that IMRT significantly reduced the mean renal doses when compared with 3D‐CRT.^9^ From 2015, IMRT for patients with gastric lymphomas have been performed in our institution.

It is well‐known that the stomach moves with respiration, and the usefulness of 4DCT has been reported in the treatment planning for stomach lymphomas.^10^ One concern using IMRT for the stomach is that the respiratory motion may cause hot or cold spots within the target. This is known as interplay effects, and interplay effects have been extensively studied for lung tumors. Rao et al. generated a five‐field IMRT and volumetric‐modulated arc photon therapy (VMAT) plan for lung tumors and showed that interplay effects can be made negligible.^11^ Several studies have shown possible advantages in the use of respiratory gating technology in combination with IMRT to reduce errors due to respiratory motion.^12^, ^13^, ^14^ Before applying IMRT for a moving target, the assessment of interplay effects should be essential as with lung tumors. Because the stomach is unique shape and moves with respiration or peristalsis, unexpected dose distributions may cause the deterioration of the target coverage. However, there are only a small number of reports that focus on interplay effects in stomach IMRT. Stomach IMRT seems to be increasing for stomach lymphomas in practice, therefore, studies on interplay effect are now required.

The aim of this study was to evaluate the impact of organ motion on volumetric and dosimetric parameters in stomach lymphomas treated with IMRT. Four‐dimensional simulation plans were created retrospectively to investigate the impact of respiratory motion on the dosimetric parameters and compared with the clinically used IMRT plans.

## 2 MATERIALS AND METHODS

### 2.1 Patients and CT imaging

This study was approved by the institutional review board of Hokkaido University Hospital. A total of ten patients who were consecutively treated with IMRT for stomach lymphomas from April 2015 to January 2018 were included in this retrospective study. Details of the patient backgrounds are shown in Table 1. To reduce the stomach volume and keep it constant during the course of treatment, the patients were told to eat nothing for at least six hours before the planning CT scan and before every treatment fraction. At first, CT scanning under free‐breathing was conducted using a CT simulator consisting of a scanner (Optima CT580 W; GE Healthcare, Waukesha, WI, USA), a flat patient table, and precise laser localizers. We term these images as free‐breathing CT images (fb3DCT) in this study. Coinciding, 4DCT images were also recorded using a surface monitoring system to detect respiration motion (RPM system, Varian Medical Systems, Palo Alto, CA, USA). The acquired 4DCT images were reconstructed into 10 sets of 3DCT images according to ten phases of the respiratory cycles using the RPM system. These sets of 3DCT images are termed 3DCT_r%_ where the r represents the proportion of time in a respiratory cycle. A representative respiratory cycle was determined with the RPM system and divided into 10 equal phases so that r = 0, 10, 20, …, and 90 for a 3DCT_r%_. As a result, there were one fb3DCT image and ten 3DCT_r%_ images for each transaxial slice. The slice thickness of a CT image for contouring was 2.5 mm both for the fb3DCT and 3DCT _r%_ images.

**Table 1:** Patient backgrounds

No	Histology	Dose (Gy)	Fraction	CTV volume (cm^3^)	PTV volume (cm^3^)	Respiratory period (Second)	Average beam‐on time per segment^a^ (Second)
1	Follicular	30.0	20	255.20	1694.85	7.2	1.3
2	MALT	30.0	20	150.17	1084.07	6.8	1.9
3	MALT	30.0	20	303.25	1590.54	3.7	1.2
4	MALT	30.0	20	284.63	1181.19	3.5	1.2
5	MALT	30.0	20	293.78	1718.86	4.5	1.3
6	DLBCL	40.5	27	273.55	1458.89	3.9	1.2
7	MALT	30.0	20	272.77	1442.19	2.7	1.2
8	MALT	30.0	20	220.32	1348.28	5.0	1.6
9	MALT	30.0	20	260.26	1726.42	3.3	1.3
10	MALT	30.0	20	427.77	1770.20	3.5	1.3

CTV, clinical target volume; DLBCL, diffuse large B‐cell lymphoma; MALT, mucosa‐associated lymphoid tissue; PTV, planning target volume.

a segmental fields formed by the MLC.

### 2.2 Radiation treatment planning

The IMRT plans were generated with the radiation treatment planning system (TPS): Pinnacle^3^ v9.0 (Philips Medical Systems, WI, USA). Each 3DCT_r%_ was fused with the fb3DCT on the TPS. This image fusion was automatically conducted based on the patient bone structure using a function available in the TPS. We term a set of fusion images which consists of 10 fused CT images at the same table position, as fus3DCT. Thus, there is one fus3DCT for one transaxial CT slice. At first, contouring of the targets and OAR was conducted on the fb3DCT. The gross tumor volume (GTV) was not determined because no obvious tumor was visible in the CT images. The clinical target volume (CTV) was contoured as the whole stomach according to the RTOG contouring guidelines.^15^ Considering interfractional uncertainty, the internal target volume (ITV) was contoured by assuming a 1.5 cm margin to the CTV routinely. Because a 1.5 cm margin was sometimes inadequate in case of the large respiratory motion, we modified the ITV to cover the stomach in the 3DCT_r%_ image of all respiratory phase. The planning target volume (PTV) was contoured by expanding a 0.5 cm margin to the ITV considering set‐up errors. The dose‐prescription was 30 Gy covering 95% of the PTV in 20 fractions for follicular lymphomas or MALT lymphomas and 40.5 Gy in 27 fractions for DLBCL. In this report, the clinically used IMRT plan, in which fus3DCT was used, was termed a F3D plan.

### 2.3 Simulation planning

**Figure 1:**
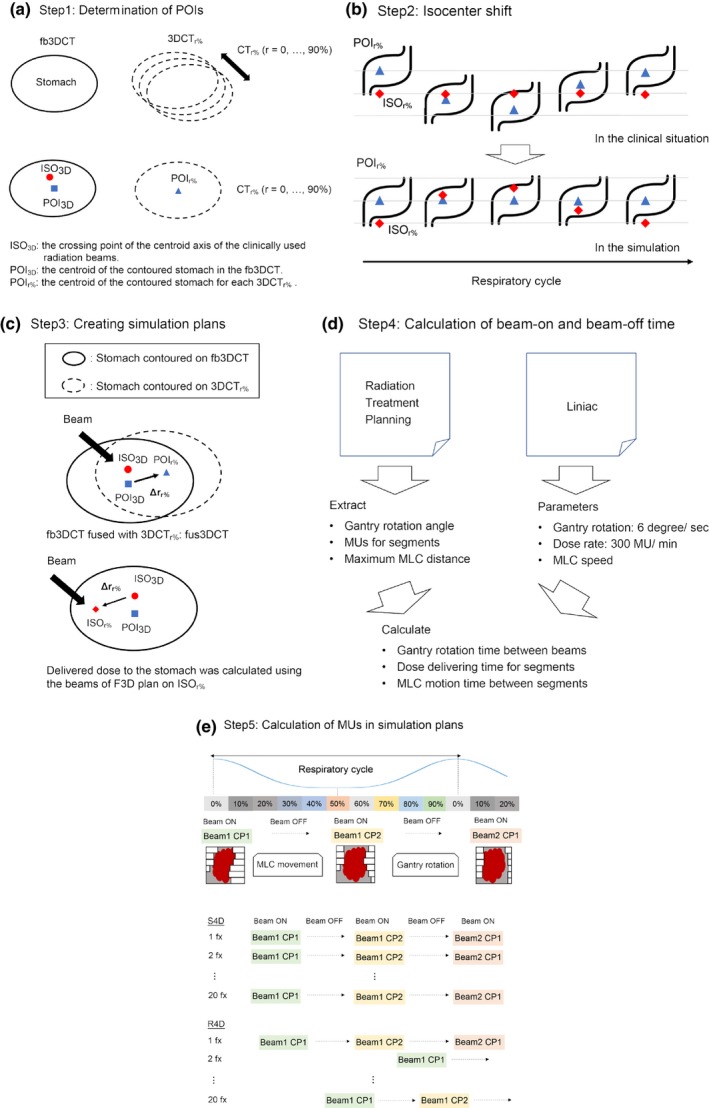
Illustration of the method used in this simulation. Details are shown in "Simulation planning" subsection of Materials and Methods section. fb3DCT, free‐breathing three‐dimensional CT; 3DCT_r%_, reconstructed 3DCT from 4DCT scan at a r% phase of a respiratory cycle; fus3DCT, fusion image set of the 3DCT_r%_ fused with fb3DCT; CP, control point; ISO, isocenter; MLC, multileaf collimator; MU, monitor unit; POI, point of interest; R4D, Random 4D simulation; S4D, Simple 4D simulation

To assess the impact of the organ motion on the dose distributions, we conducted a simulation study on the virtual coordinates in the TPS in this study. This simulation planning was not used in the actual treatment but created for analysis only in this study. The simulation method is shown in Fig. 1.

The isocenter of the fb3DCT, which is the central axis of the clinically used radiation beam, is named ISO_3D_ in this study_._ The centroid of the contoured stomach in fb3DCT was also defined as POI_3D_. We contoured all stomachs in 3DCT_r%_ images of ten respiratory phases (r = 0, 10, 20, …, 90) in the respiratory cycle for this simulation study and the centroid of the contoured stomach was similarly defined as POI_r%_ for each 3DCT_r%_ (step 1).

To simulate the organ motion of the stomach relative to the treatment beams on the TPS, the situation was modeled by using a corresponding isocenter shift as reported in previous studies.^16^, ^17^ To summarize, the stomach motion was reproduced on the TPS by shifting the isocenter and beams three‐dimensionally in the opposite direction of the actual organ motion. We assumed that the change in the depth from the entrance at the skin surface to the stomach did not change significantly and used the beam parameters for the F3D plan in this analysis. We also assumed that the stomach moves as a rigid organ during respiration and did not consider deformation during a respiratory cycle in this study (step 2).

To estimate the dose delivered to the stomach in the 10 respiratory phases of a cycle, differences between POI_r%_ and POI_3D_ (Δ**r**_r%_ **= r**(POI_r%_) − **r**(POI_3D_)) were measured on the same co‐ordinates on the fus3DCT. The delivered dose to the stomach at an r% phase was calculated virtually using the beam parameters for the F3D plan and the assumed isocenter at the r% phase, ISO_r%._ which is calculated by **r**(ISO_r%_) = **r**(ISO_3D_) ‐ Δ**r**_r%_, rather than the ISO_3D_ (step 3).

In the actual treatment, step‐and‐shoot IMRT, 6 MV X‐ray beams from seven static, different directions were used with Clinac iX linear accelerators (Varian Medical Systems, Palo Alto, CA, USA). We extracted the gantry angle of each static direction, monitor units (MU) for each control point (CP), and the maximum multileaf collimator (MLC) distances between sequential CP from the F3D plan. Average number of CP per beam was 20.25 (range: 6–36). Each CP represents the segmental field made by the MLC during the delivery of the IMRT. To calculate the beam‐on and beam‐off durations, a gantry rotation speed of 6 degree/s and a dose rate of 300 MU/min were used. To calculate the duration of the MLC motion between CP, the MLC speed was determined from the log data in the Clinac iX linear accelerators. Using these parameters, we estimated the time for the gantry rotation between two sequential beams, the dose delivery times for CP, and the duration of the MLC motion between CP. The average respiratory cycle in each patient was extracted from the log data in the RPM system (step 4).

We investigated the dosimetric effects of the respiratory motion during IMRT by two simulation methods. One, we assumed that beam‐on timing is simply fixed at a phase in the average respiratory cycle without any gating or training for respiration and call this a simple four‐dimensional (S4D) simulation. The second method is a simulation plan assuming random beam‐on timing relative to a respiratory cycle and this is called a random four‐dimensional (R4D) simulation. In clinical situations, radiation beam‐on timing relative to the patient respiratory cycle is not considered. Therefore, the beam‐on timing is randomly distributed during free breathing. The R4D simulation is a simulation of the IMRT under free breathing without any gating or training for respiration. In the S4D simulation for a specific respiratory phase, we investigated the effect of four different beam‐on timings (0%, 25%, 50%, and 75%) relative to the respiratory cycle. S4D simulations where the beam delivery starts at 0%, 25%, 50%, or 75% in the respiratory cycle, were indicated as S4D‐0%, 25%, 50%, and 75%, respectively. The average of these four values was indicated as S4D‐average. We also calculated the beam delivery time for each respiratory phase at every CP. With the clinically used dose rate (300 MU/ min), the delivered MU for each respiratory phase in the simulation plan was computed. We assumed that the gantry rotation between beams, dose delivery, and MLC motion were continuous, which is like that of the Rao's article.^11^ In the R4D simulation, the beam delivery time was calculated assuming that the beam‐on timing was randomly selected within the respiratory cycle. From the obtained beam delivery time and dose rate, we calculated MU in each CP for every respiratory phase with multiplying beam delivery time by dose rate. At this time, total MU in simulation plans are equal to the clinical plan. This independent calculation was repeatedly performed 20 times in nine patients and 27 in another in S4D and R4D simulations. These numbers are same as the clinically prescribed number of fractions (shown in Table 1) to compare dose distributions of simulation plans with clinically used IMRT plan. Finally, the summed MU for each CP was inputed into the TPS. In this way, total composite dose distributions were obtained (step 5).

### 2.4 Evaluation and statistical analysis

The stomach motion was evaluated as the POI_r%_ motion of 3DCT_r%_ images in the anterior‐posterior (AP), left‐right (LR), and superior‐inferior (SI) directions. The respiratory amplitude was defined as the maximum difference among POI_r%_ positions in each direction. Furthermore, the stomach volume in each of the 3DCT_r%_ images was also measured to evaluate the change in stomach volume in a respiratory cycle. The percentage of stomach volume in 3DCT_r%_ compared to the average stomach volume over the ten 3DCT_r%_ sets was calculated in each patient.

Several dosimetric parameters of the stomach on the fb3DCT were evaluated. Here, Dx is defined as the radiation dose administered to the X% volume of the region involved. The V_p_ is the percentage of target volume covered by the prescribed dose. The homogeneity of the target was evaluated by a homogeneity index (HI) defined as (D_2_‐D_98_)/ D_50_ according to International Commission on Radiation Units & Measurements (ICRU)‐83.^18^ The dosimetric parameters of the S4D simulation were averaged over four different beam‐on timings. A dose grid of 2 mm was used for the calculations. The Wilcoxon matched‐pair signed rank test was used to compare dosimetric parameters. A *P*‐value < 0.05 was considered to be significant.

## 3 RESULTS

### 3.1 Stomach volume and motion

**Figure 2:**
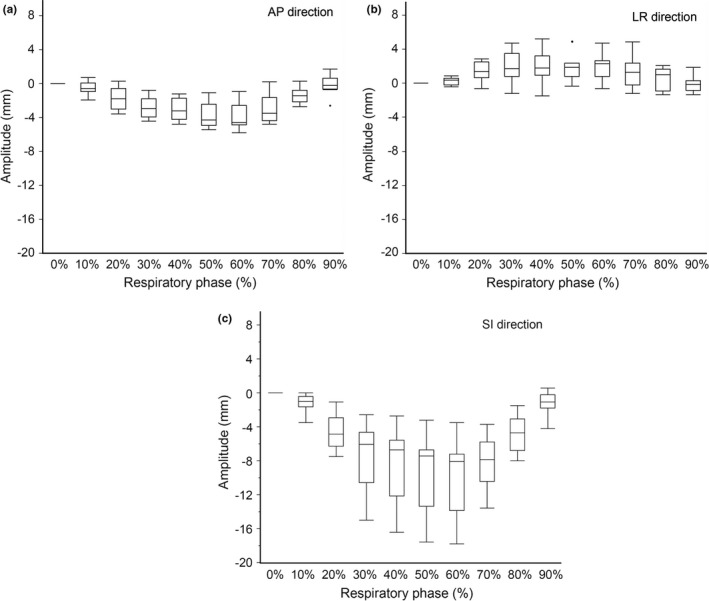
Stomach motion of 3DCT_r%_ images, (a) AP, (b) LR and (c) SI direction. The stomach motion is evaluated by POI_r%_ in 3DCT_r%_. The coordinates of the stomach centroid in the 0% CT image was set as zero. Boxes indicate the interquartile range from the 25 to 75‰. Median and outliers are shown as horizonal lines within the box and filled circles, respectively

The stomach amplitude among the 3DCT_r%_ images is shown in Fig. 2. The coordinates of the stomach centroid in the CT_0%_ image (POI_0%_) was set as zero. The average respiratory amplitude ± 1SD in the 10 patients were 4.1 ± 1.4, 2.9 ± 1.3, and 10.1 ± 4.5 mm in the AP, LR, and SI directions, respectively. The largest respiratory motion was observed in the SI direction. The respiratory amplitude in the SI direction varied considerably among the 10 patients (range: 3.7–16.9 mm).

**Figure 3:**
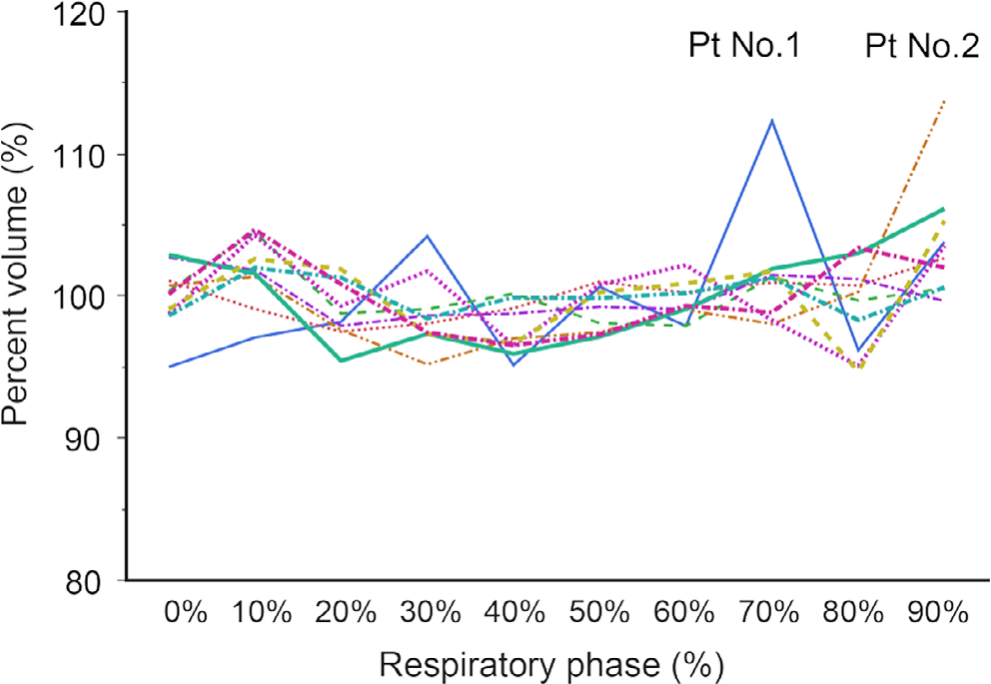
Percent volume of stomach in 3DCT_r%_. The stomach contouring is conducted for every 3DCT_r%_ image and its volume was calculated. The colored line shows values for individual patients. The vertical axis shows percent volume of the stomach to the averaged stomach volume over ten sets of 3DCT_r%_ images

Mean stomach volume with 1SD was 273.7 ± 68.5 cm^3^. The percentage (ratio of the mean value) of the volume of the stomach in each 3DCT_r%_ image is shown in Fig. 3. The percentage (%) of the stomach volume in each of the respiratory phases compared to the mean volume varied generally within an around ± 5% range in most of the patients (Fig. 3). Larger than 10% volume fluctuations were found in the CT_70%_ image of patient No.1 (+12.3%) and CT_90%_ in patient No.2 (+13.7%).

### 3.2 Dose distributions

The median (range) of the HI, the volume percentage of the stomach covered by the prescribed dose (V_p_ (%)), and D_99_ of CTV in the F3D plan, S4D simulations, and R4D simulation are shown in Table 2.

**Table 2:** Homogeneity index (HI), V_p_ (%), and D_99_ of the CTV in simulated plans

	HI	V_p_ (%)	D_99_ (Gy)
F3D plan	0.045 (0.036–0.080)	99.71(97.59–99.93)	30.30 (30.10–30.67)
S4D‐0%^a^	0.076 (0.056–0.015)	92.39 (80.11–99.61)	29.65 (28.60–30.30)
S4D‐25%^a^	0.080 (0.068–0.137)	94.83 (70.46–98.04)	29.70 (28.60–30.00)
S4D‐50%^a^	0.079 (0.054–0.126)	96.15 (80.93–99.51)	29.45 (28.80–30.30)
S4D‐75%^a^	0.075 (0.052–0.165)	93.77 (82.57–99.70)	29.55 (28.89–30.40)
S4D‐average^a^	0.078 (0.065–0.141)	92.12 (82.31–99.14)	29.58 (28.73–30.20)
R4D	0.052 (0.035–0.127)	97.24 (92.85–100)	30.05 (28.80–30.40)
p‐value			
F3D plan vs			
S4D‐0%	0.002	<0.001	<0.001
S4D‐25%	0.001	<0.001	<0.001
S4D‐50%	0.002	0.001	0.002
S4D‐75%	0.002	<0.001	0.002
S4D‐average	0.001	<0.001	<0.001
R4D	0.762	0.014	0.012

Values are presented as the median (range). HI is calculated as (D_2_‐D_98_)/D_50_ in CTV. V_p_ is volume percentage of stomach covered by the prescribed dose. D_99_ is defined as radiation dose given to 99% volume of the region involved. The Wilcoxon matched‐pair signed rank test was used to compare dosimetric parameters.

CTV, clinical target volume; F3D plan, the clinically used IMRT plan; HI, homogeneity index; R4D, random 4D simulation; S4D, simple 4D simulation.

a S4D‐0, 25, 50, and 75% indicate S4D simulations where the beam delivery starts at 0, 25, 50, or 75% in the respiratory phase, respectively. S4D‐average is the average of these four values.

The median (range) of HI was 0.045 (0.036–0.080) in the F3D plan. The median of HI in four S4D simulations where the beam‐on timings are different (0, 25, 50, and 75%) and their average showed significantly poorer homogeneity than the F3D plan (*P *≤ 0.002). However, the median (range) of HI was 0.052 (0.035–0.127) in R4D and there were no statistically significant differences in HI between the F3D plan and R4D (*P *= 0.762).

The median (range) of V_p_ (%) was 99.71 (97.59–99.93) in the F3D plan. The median of V_p_ in the four S4D simulations and their average showed significantly poorer coverage than the F3D plan (*P *≤ 0.001). The median of V_p_ in the R4D simulation was 97.24 (92.85–100), also a significantly poorer coverage than in the F3D plan (*P *= 0.014).

**Figure 4:**
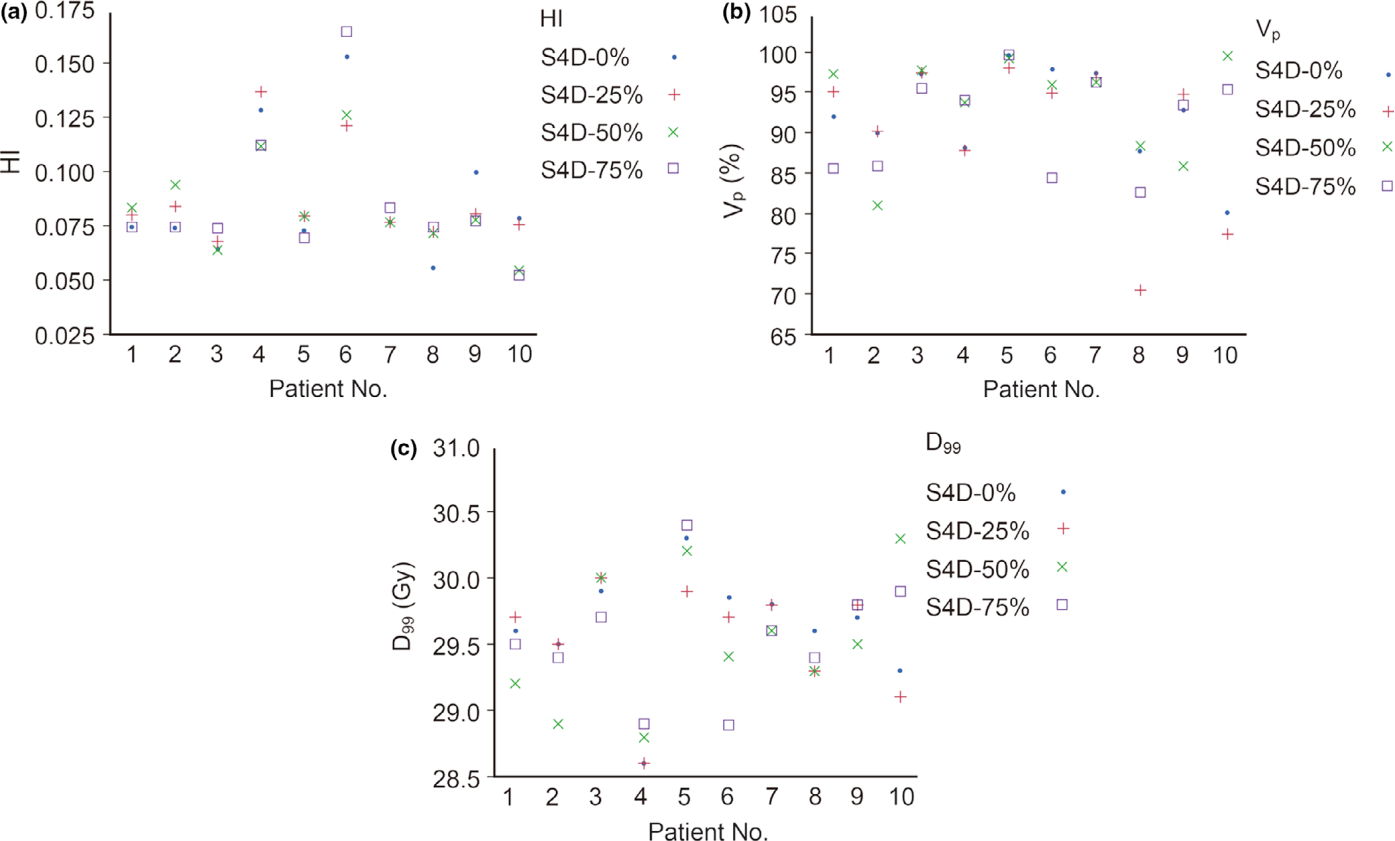
Dosimetric parametes in the S4D simulations. S4D‐0% means the S4D simulation at the start of beam delivery, at 0% in the respiratory phase. (a) HI (homogeneity index) is calculated as (D_2_‐D_98_)/D_50_ in CTV. (b) V_p_ is the volume percentage of the stomach covered by the prescribed dose. (c) D_99_ is the radiation dose given to 99% of the volume of the region concerned

For the prescribed dose, all patients received 30.0 Gy, except one (patient No. 6) who had 40.5 Gy prescribed. To normalize this in the calculation of the median (range) of D_99_ (Gy), the dose distribution of patient No. 6 was rescaled assuming a 30 Gy dose rather than the 40.5 Gy. As a result, the median (range) of D_99_ was 30.30 (30.10–30.67) Gy in the F3D plan. The median of D_99_ in the four S4D simulations and their average was significantly lower than in the F3D plan (*P *≤ 0.002). The median of D_99_ in R4D simulations was 30.05 (28.80–30.40) Gy and also significantly lower than in the F3D plan (*P *= 0.012). However, the difference was 0.25 Gy (<1% of the prescribed dose) between R4D and F3D plans and the median D_99_ in the R4D plan exceeded the prescribed dose. In the S4D simulation, the HI, V_p_ (%), and D_99_ of CTV varied among the patients, depending on the beam‐on timing in the respiratory cycle (0%, 25%, 50%, and 75%) (Fig. 4).

## 4 DISCUSSION

Dosimetric effects of respiratory motion in lung cancers has been extensively studied,^19^, ^20^, ^21^ however, we were unable to locate published articles for the stomach. In this report, we present the respiratory motion of the stomach and its impact on dosimetric parameters in IMRT plans. This simulation study was conducted based on clinically delivered IMRT plans, and the respiratory motion of the stomach was extracted from 4DCT images for each of the patients.

Our simulation study has shown that the amplitudes of the stomach motion is largest in the SI‐direction (Fig. 2). This result is similar to previous reports. Watanabe et al. studied intrafractional gastric motion using repeated CT scans before the course of treatments.^22^ They reported that the average intrafractional motions were 4.6, 2.4, and 12.1 mm for the AP, LR, and SI directions, respectively. Wysocka et al. concluded that the median respiratory motions were 8.8, 1.7, and 16.4 mm in the AP, LR, and SI directions using inspiration and expiration CT images.^23^ When considering intrafractional stomach motions, nonrespiratory motion must also be taken into consideration. Hashimoto et al. detected the effect of cardiac motion on the motion of the upper digestive tract by implanting a fiducial marker but the effect was much smaller than that of the respiratory motion.^24^ Wysocka et al. assessed nonrespiratory stomach motions of healthy volunteers in fasting states using cine‐MRI scanning.^25^ The Wysocka study reported that the displacement from the baseline position was small, rarely exceeding absolute values of 1.1 mm, with the largest value 1.6 mm in the SI direction. From this, they concluded that the main source of intrafractional stomach motion was due to respiration. These results, including this study, suggest that interplay effects involving the stomach, if there are any, are mainly caused by the respiratory motion.

We found that volume fluctuations of the stomach were almost all within the range of ± 5%, of the average stomach volumes during a respiratory cycle in this study. Hallman et al. evaluated volume fluctuations of the liver during respiration using 4DCT and reported volume variations of less than 4%.^26^ The stomach is essentially an empty bag, and we anticipated that the volume of the stomach would vary considerably with respiration. However, the stomach volume in each of the respiratory phases varied largely only within a ± 5% range comparing to the mean volume in most of the patients. This very limited variation in stomach volume among the 3DCT_r%_ images indicates that there is little deformation of the stomach during a respiratory cycle. However, this still does not address interfractional changes in the stomach volume which was not investigated in this study. We requested only dietary control for the patients and did not evaluate the actual size and position of the stomach every day in the actual treatment.

Before considering interplay effects in IMRT, it is necessary to determine whether the internal margin adopted is sufficient to irradiate the stomach with the prescribed dose.^27^ Waghorn et al. studied the dosimetric impact of motion on step‐and‐shoot IMRT lung plans.^28^ They demonstrated that a target volume ratio covered by 95% of the prescribed dose was associated with a margin from the target to the PTV. In this study, we generally added 1.5 cm ITV margin with additional expansion considering the fus3DCT and then a 0.5 cm set‐up margin for the PTV. Here, the maximum amplitude of the stomach was estimated as 10.1 ± 4.5 mm in the SI directions, and the margin recipe used in our institution was shown to be appropriate to cover the CTV during IMRT.

We investigated interplay effects on the dose distribution by creating two kinds of virtual simulations, four S4D and a R4D simulation. The median of HI, V_p_, and D_99_ in the S4D simulations was significantly poorer than those of the F3D plan for the four different beam‐on timings in a respiratory cycle. Because the beam‐on timing is determined at one particular point in a day, the S4D simulation in which beam‐on timing was fixed essentially represents dose‐distributions in one fraction. As long as beam‐on timing is fixed, HI and V_p_ does not depend on the number of fractions. As for D_99_, it depends on the number of fractions proportionally. Physicians have to know that daily one fraction in a course of treatments have potentially significant interplay effects. Moreover, the results shown in Fig. 4 suggest that the dose distribution in the daily IMRT varies depending on the beam‐on timing in each patient. Hot or cold spots arising in S4D simulations were averaged out in the R4D simulation resulting in the smaller difference from the F3D plan in the R4D simulation. Our simulations agree with those of other reports about lung lesions that showed interplay effects to generally become weaker as the number of fractions increase.^29^, ^30^ In the comparison of V_p_ and D_99_, the R4D simulation showed a small but statistically significantly lower median value than in the F3D plan suggesting that even in the free‐breathing and multiple fractionated treatment, interplay effects affected the dose distribution in stomach IMRT. Stomach MALT lymphomas are radiosensitive malignancies and absolute dose reduction was less than 1% of the prescribed dose due to interplay effects. This result leads us to think that the clinical effect must have been minimal and stomach step‐and‐shoot IMRT is permissible even if interplay effects exist. In this study, we did not perform simulations of hypofractionated scheme. Although hypofractionated radiotherapy for stomach lymphomas seems to be uncommon, we predict that it will attenuate the interplay effects as far as the beam‐on timing is at random and the dose rate is not too high as shown in previous article.^11^ However, since recent linear accelerators produce x‐ray at very high dose rate, the beam delivery time can become shorter. As a result, the beam‐on timing in respiratory cycle become more influential and the interplay effects can be much larger than we have seen in this study.

The limitations of our study include the following. It is a simulation study based on several assumptions and not measuring actual doses in the body. Respiratory motion was detected by a surface monitoring device and may not be consistent with the internal organ motion. The effect of organ motion was simulated by changing the isocenter position in the TPS which might have been affected the dosimetric parameters. Moreover, we did not calculate doses to OAR since it was not easy to calculate interplay effects to other organs using above simulation methods. Because the movements of OAR are expected to be different from the stomach, we have to evaluate their own respiratory motions using 4DCT images and shift beams oppositely for each organ. The interfractional change in stomach volume and position was not accounted for in the 4D simulation as mentioned above. We have not investigated the possible effect of respiratory gating in the IMRT for the stomach. Considering the significant interplay effects in S4D simulation, the synchronization of respiratory phase and beam‐on timing should be used with care in the IMRT for the stomach. These uncertainties are still remaining and should be investigated further.

## 5 CONCLUSIONS

We have demonstrated the respiratory motion and the volume change in the stomach volume using 4DCT images of ten patients. Our 4D simulations suggested that interplay effects did not affect HI significantly but affected V_p_ and D_99_ even though IMRT for the stomach was delivered in free breathing and multiple fractions. However, the magnitude of the dose reduction was small. Further studies will be required to reduce interplay effects of stomach.

## CONFLICTS OF INTEREST

The authors declare that there are no conflicts of interest associated with this manuscript.
